# Extensive Aortic Thromboembolism in a Patient With Erdheim-Chester Disease: A Case Report

**DOI:** 10.3389/fcvm.2022.882817

**Published:** 2022-05-13

**Authors:** Jiangping He, Xin Fang, Xianfeng Zhang, Kuang Chen, Jiao Huang

**Affiliations:** ^1^Department of Rheumatology, Affiliated Hangzhou First People’s Hospital, School of Medicine, Zhejiang University, Hangzhou, China; ^2^Department of Vascular Surgery, Affiliated Hangzhou First People’s Hospital, School of Medicine, Zhejiang University, Hangzhou, China; ^3^Department of Hematology, Affiliated Hangzhou First People’s Hospital, School of Medicine, Zhejiang University, Hangzhou, China

**Keywords:** aortic thromboembolism, Erdheim-Chester disease, non-Langerhans cell histiocytosis, splenic infarction, lipogranulomatosis

## Abstract

**Background:**

Erdheim-Chester disease (ECD) is a rare disease that affects multiple systems and is characterized by non-Langerhans cell histiocytosis. Classic clinical signs include long bone infiltration, central nervous system involvement, diabetes insipidus, and sheathing of the entire aorta. However, thrombosis is not recognized as a typical cardiac manifestation of ECD. Here, we report the case of an ECD patient with extensive arterial thrombus formation and embolism in several sections of the aorta.

**Case:**

A 36-year-old woman was admitted due to recurrent fever and left finger cyanosis for 20 days. Laboratory tests revealed that her C-reactive protein and interleukin-6 levels were significantly elevated. Thoracic computed tomographic angiography (CTA) revealed thrombosis from the aortic arch to the left subclavian artery accompanied by severe stenosis of the left subclavian artery. Abdominal CTA revealed splenic infarction due to splenic artery embolism and thrombus formation in multiple abdominal arteries. She underwent emergent arterial thrombectomy. During hospitalization, she complained of polyuria. The desmopressin test and pituitary magnetic resonance imaging findings suggested diabetes insipidus. Furthermore, positron emission tomography-computed tomography and bone emission computed tomography showed long bone impairment, and pathological examination of the bone samples confirmed ECD. Steroids and tocilizumab were selected as the initial therapies; however, thrombosis continued to develop. After replacement of tocilizumab with interferon-α, her condition became stable.

**Conclusion:**

Although extremely rare, fatal thrombosis may be a significant cardiovascular manifestation of ECD.

## Introduction

Erdheim-Chester disease (ECD) is a rare disease that refers to a series of clinical manifestations caused by non-Langerhans cell histiocytosis. Since it was first described by anatomopathologist Erdheim and his student Chester in 1930, no more than 2,000 cases have been reported worldwide.

ECD can lead to fatal outcomes, especially when it affects multiple systems. The main clinical signs include bone impairment (79%), diabetes insipidus (48%), coated aorta (40%), and hairy kidney appearance on computed tomography (CT) scan (63%) (1). Only one case was reported wherein ECD was associated with arterial thromboembolism (2).

Herein, we report a rare case, in which the patient was diagnosed with ECD following extensive aortic thrombosis and splenic infarction.

## Case Description

A 36-year-old woman was admitted due to recurrent fever and left finger cyanosis for 20 days. She had undergone a cesarean section 26 days prior. The patient also complained of abdominal pain and nausea. Body temperature was 38.3^°^C, and other vital signs were stable. Physical examination showed that the fingers of her left hand were pale and her skin temperature was much lower than that on the right side. A murmur in the left subclavian artery was auscultated.

## Diagnostic Assessment and Therapeutic Intervention

### Diagnostic Assessment

**FIGURE 1:**
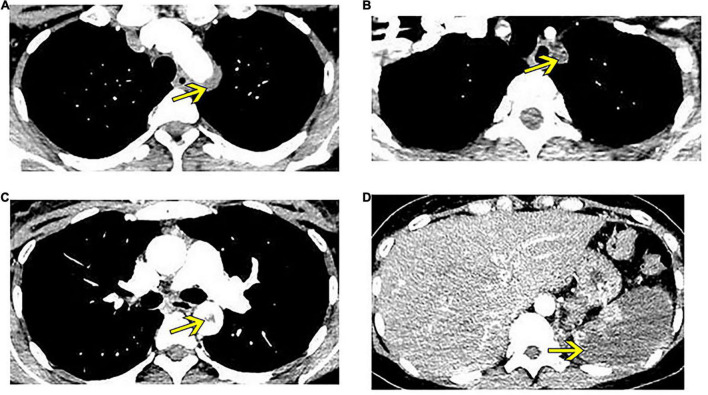
Enhanced computed tomography shows thrombus formation in several arteries (yellow arrows). **(A)** Thrombus at the aortic arch. **(B)** Thrombus crosses to the left subclavian artery, leading to embolism of this artery. **(C)** Thrombus at the thoracic aorta. **(D)** Splenic infarction.

Routine blood test showed a white blood cell (WBC) count of 12.7 × 10^9^/L, C-reactive protein (CRP) level of 60.2 mg/L, and erythrocyte sedimentation rate (ESR) of 36 mm/h. Blood biochemistry was within the normal range, and serological examinations, including autoimmune antibodies and antiphospholipid antibodies, were negative. CTA of the thoracic and abdominal aorta showed thrombosis starting from the aortic arch to the left subclavian artery, accompanied by severe stenosis of the left subclavian artery, diffuse splenic infarction due to embolism of the splenic artery, and thrombosis in several arteries, including the common hepatic artery, left common iliac artery, and proximal to the femoral artery (Figure 1). The patient was diagnosed with extensive arterial embolism secondary to unknown reasons and presented a risk of thrombus drop, which would lead to embolism in other vital organs. Therefore, emergent catheter-based arteriography and thrombectomy in the left subclavian artery was performed, while balloon block in the superior mesenteric and renal arteries was adopted to prevent thrombus drop.

Surgery was successful; however, the patient’s fever persisted. Laboratory examinations revealed elevated levels of blood CRP at 108 mg/L, serum sodium at 163 mmol/L, and serum interleukin-6 (IL-6) at 198.69 pg/mL. The blood culture results were negative. She also complained of thirst and polyuria, and her urine volume collected for 24 h was 7,000 mL. Pituitary enhanced magnetic resonance imaging (MRI) revealed nodular thickening of the pituitary stalk (Supplementary Figure 1) and the desmopressin test result was positive, which were supportive of a diagnosis of diabetes insipidus. Sex hormones were assessed, and the levels of follicle-stimulating hormone and luteinizing hormone were found to be extremely low, suggesting anterior pituitary hypofunction. Positron emission tomography (PET)-CT suggested inflammatory-reactive changes in superficial lymph nodes, slight interstitial pneumonia, and most importantly, multiple bone changes in the scanned area. For further confirmation, bone emission computed tomography (ECT), knee CT, and knee MRI were performed, which showed diffuse bone infiltration in bilateral distal femurs and proximal tibias (Supplementary Figures 2A–C).

**FIGURE 2:**
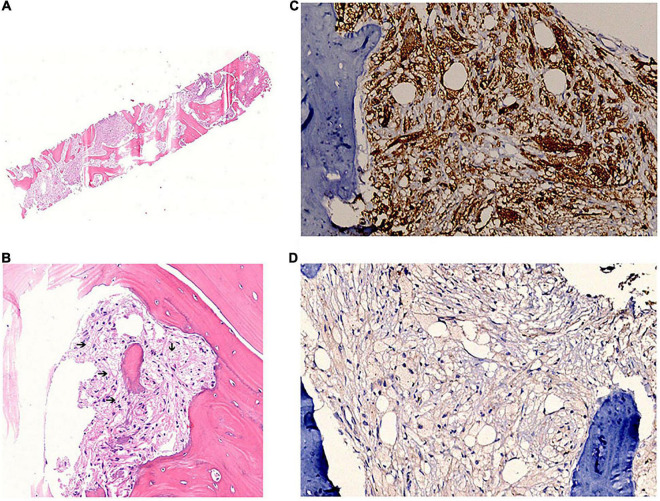
Bone sample pathology and immunohistochemistry confirms the diagnosis of ECD. **(A)** Hematoxylin and eosin staining of the bone sample. **(B)** Foamy cells surrounded by fibrosis (black arrows). **(C)** CD68 staining is positive. **(D)** CD1a staining is negative.

A multidisciplinary discussion was conducted, and considering the pituitary lesion and the long bone infiltration, the diagnosis of ECD was proposed. Further examination was performed. Brain enhanced MRI revealed thickening of cerebral falx, which is a typical change in ECD (Supplementary Figure 3), while the cardiac MRI examination did not show a coated aorta. Bone puncture was finally approved, and the results showed a large number of foamy cells surrounded by fibrosis and few multinucleated giant cells (Figure 2). Immunohistochemistry (IHC) revealed CK [−], CD68 [+], CD163 [+], Langerin [−], CD1a [−], and S100 [+], in accordance with the changes in ECD. In addition, a circulating *BRAF*^*V*600*E*^ mutation was identified, further confirming the diagnosis of ECD.

### Therapeutic Intervention

Upon diagnosis, the following medications were administered: methylprednisolone 40 mg intravenously twice a day, rivaroxaban 20 mg and aspirin 100 mg orally every day as anticoagulant therapy, and Minirin (desmopressin) 0.05 mg orally three times a day to reduce the urine volume. Her body temperature returned to normal immediately after treatment. Because of the elevated IL-6 levels, we used tocilizumab 8 mg/kg to suppress the inflammatory state, and the dosage of methylprednisolone was gradually reduced to 20 mg orally per day as maintenance. A repeat blood test performed 1 week after therapy showed normal WBC count, CRP, ESR, and IL-6 levels.

**FIGURE 3:**
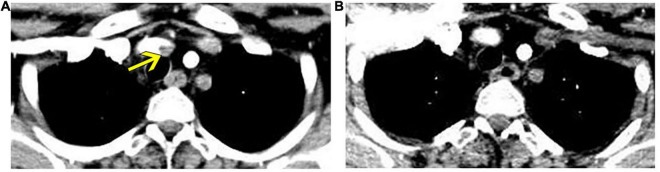
Enhanced thoracic computed tomography reveals fresh thrombus formation in the brachiocephalic trunk, which regressed after interferon-α treatment. **(A)** Fresh thrombus in the brachiocephalic trunk (yellow arrow). **(B)** Regression of thrombus in a reviewed computed tomography after 2 months of interferon-α treatment.

However, 1 month later, when the patient was admitted for review, enhanced CT of the thoracic aorta showed fresh thrombus formation in the brachiocephalic trunk (Figure 3A). Laboratory tests showed that CRP was elevated at 28 mg/L, although there were no symptoms according to the patient. This CT finding suggested progression of the disease; therefore, tocilizumab administration was discontinued and replaced by interferon-α 60 μg *via* subcutaneous injection three times every week. After 2 months of treatment, repeat CT scan showed disappearance of the thrombus in the brachiocephalic trunk (Figure 3B), and her vital signs and laboratory test results returned to normal.

## Discussion

ECD is a rare disease first reported in 1930. The diagnosis of ECD is based on typical clinical manifestations, radiological findings, and histological results, excluding other mimics. In our case, the patient had diabetes insipidus, long bone infiltration, and meningeal thickening. Bone histopathology demonstrated foamy histiocytes admixed with fibrosis, and IHC confirmed non-Langerhans histiocytosis. Although the *BRAF*^*V*600*E*^ mutation was not detected in her bone sample, which may be attributed to decalcification during sample preparation, the circulating *BRAF*^*V*600*E*^ mutation was identified. After specialist treatment, the patient’s symptoms resolved, and the laboratory results became normal. Collectively, these findings established the diagnosis of ECD.

Among the clinical features of ECD, thrombosis is extremely rare, with only one case reported. In our patient, extensive thrombosis was observed in different sections of the aorta, including the aortic arch, left subclavian artery, aorta ventralis, common hepatic artery, and the splenic artery. It is regrettable that we did not obtain the thrombus sample for physiological examinations to elucidate the relationship of the thrombus and ECD. Nevertheless, the onset of thrombosis formation was in accordance with the activity of ECD verified by blood tests, radiological findings, and pathological results. Other possible causes of thrombosis, such as autoimmune diseases and malignancies, were also excluded. Moreover, during the course, the patient developed progression of thrombosis, accompanied by an elevated CRP level; after changing her therapy to interferon-α, the CRP levels returned to normal and neonatal thrombosis disappeared. These findings suggested that in our patient, thrombus formation was closely related to the activity of ECD, and the treatment of ECD was also effective for treating thrombosis, indirectly suggesting the possibility that thrombosis was formed secondary to ECD.

ECD can result in fatal outcomes, and spontaneous regression is rare. In a retrospective review, 10.1% of patients with ECD had overlapping myeloid neoplasms (3). Therapeutic regimens for ECD include immunosuppressive therapy (interferon-α, IL-6 inhibitor, and IL-1 inhibitor), nucleoside analog (cladribine), and targeted therapy (vemurafenib and cobimetinib). Interferon-α is recognized as the best initial choice for ECDs (4). In recent years, promising advances have demonstrated that BRAF and MEK inhibitors have robust efficacy, especially in multi-system and refractory ECD (5, 6). In mild cases, biological agents, such as anakinra (7) tocilizumab (8), infliximab (9), or steroids plus sirolimus (10) can be used. In our case, we started treatment with steroids and tocilizumab; however, the disease was not well controlled. After adjustment with interferon-α, the patient achieved remission. With the advent of targeted therapies, even severe manifestations of ECD have become a chronic, rather than fatal, illness (11). In our patient, thrombosis was the major and unusual manifestation. In the subsequent follow-up examinations, despite the classical lesion of ECD, we should especially consider thrombosis formation. If no further fatal thrombus is formed after treatment, the longevity of the patient may be optimistic. Given that interferon treatment is relatively long-term and may have adverse effects, such as fatigue and depression, targeted therapy may be attempted in the future.

## Patient Perspective

ECD is a rare but fatal disease; therefore, early and correct diagnosis is important for improving patient outcomes. According to reported cases and studies, thrombosis is not recognized as a clinical feature of ECD. However, in our case, extensive thrombosis and embolism of vital arteries produced the main life-threatening symptoms of the patient. Although we have no direct evidence on the relationship between thrombosis and ECD, the patient’s symptoms resolved with interferon therapy and her thrombosis has been stable. This case suggests that the understanding of ECD is not comprehensive; fatal thrombosis may be a significant cardiovascular manifestation of ECD, and other clinical signs may emerge if we continue to explore this condition.

## Data Availability Statement

The original contributions presented in the study are included in the article/Supplementary Material, further inquiries can be directed to the corresponding author/s.

## Ethics Statement

Written informed consent was obtained from the individual(s) for the publication of any potentially identifiable images or data included in this article.

## Author Contributions

JH, KC, JPH, and XF contributed in patient diagnosis, treatment, and follow-up. JPH collected the data and drafted this manuscript. JH and XZ revised the final version of the manuscript. All authors agreed to be accountable for the content of the work.

## Conflict of Interest

The authors declare that the research was conducted in the absence of any commercial or financial relationships that could be construed as a potential conflict of interest.

## Publisher’s Note

All claims expressed in this article are solely those of the authors and do not necessarily represent those of their affiliated organizations, or those of the publisher, the editors and the reviewers. Any product that may be evaluated in this article, or claim that may be made by its manufacturer, is not guaranteed or endorsed by the publisher.
