# Supplementary material for: Extensive Aortic Thromboembolism in a Patient With Erdheim-Chester Disease: A Case Report
Source: Front Cardiovasc Med. 2022 May 13;9:882817. doi: 10.3389/fcvm.2022.882817 (PMC9139267; doi:10.3389/fcvm.2022.882817)
Supplement: Supplementary file 1 [file Data_Sheet_1.docx]

Supplementary Material

## Supplementary Figures


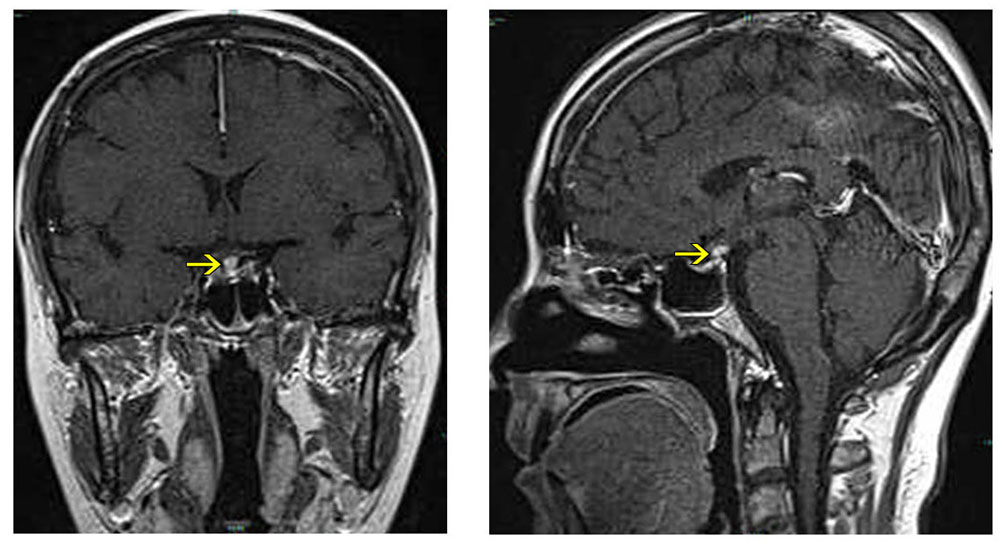


**Supplementary Figure 1.** Pituitary enhanced magnetic resonance imaging (MRI) reveals nodular thickening of the pituitary stalk.


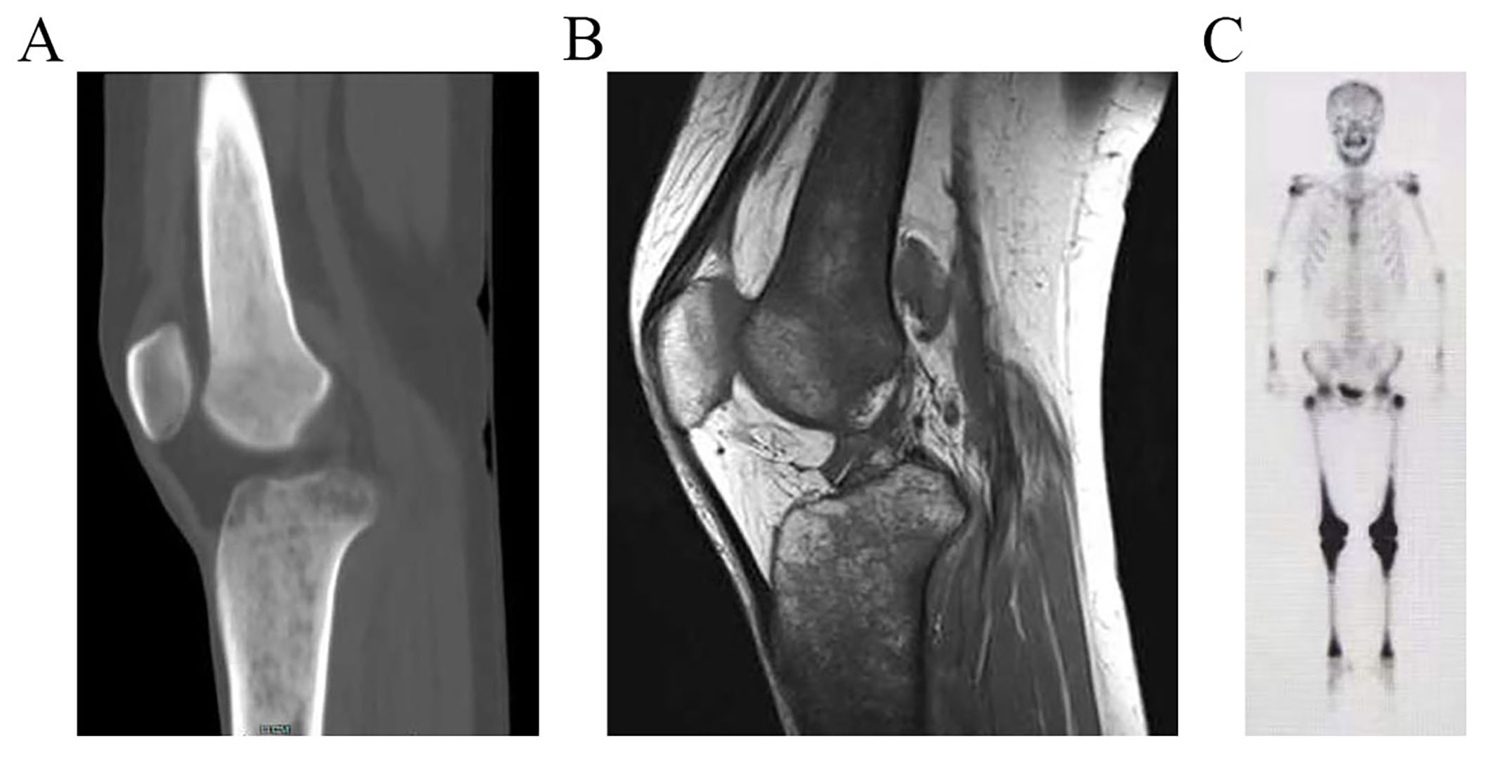


**Supplementary Figure 2.** Bone imaging revealed multiple long bone infiltrations. **(A)** Computed tomography scan of the right knee. (**B**) Magnetic resonance image of the right knee. **(C)** Bone emission computed tomography image.


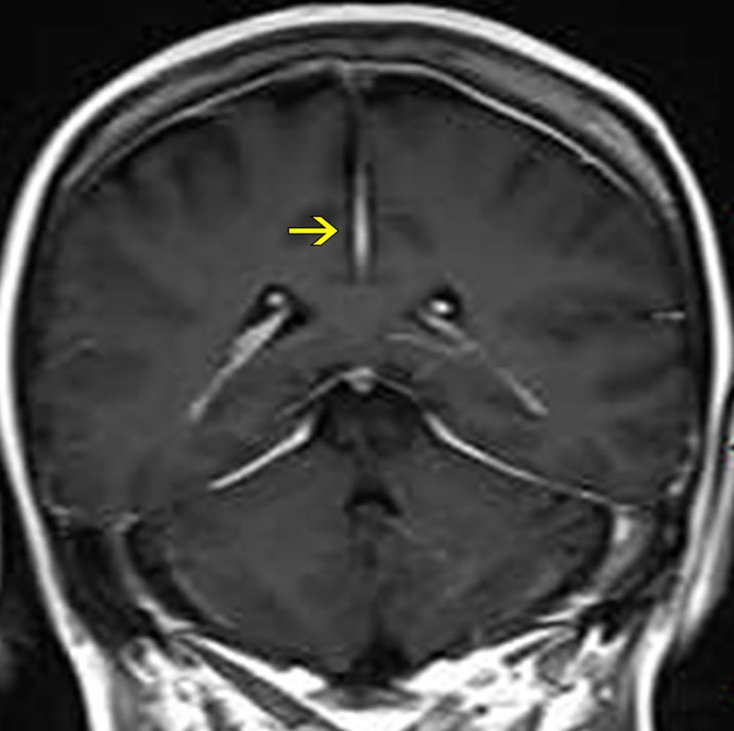


**Supplementary Figure 3.** Brain enhanced MRI revealed thickening of cerebral falx.
